# The Effect of Carbohydrates and Bacteriocins on the Growth Kinetics and Resistance of *Listeria monocytogenes*

**DOI:** 10.3389/fmicb.2018.00347

**Published:** 2018-03-01

**Authors:** Danielle R. Balay, Michael G. Gänzle, Lynn M. McMullen

**Affiliations:** Department of Agricultural, Food and Nutritional Science, University of Alberta, Edmonton, AB, Canada

**Keywords:** carnocyclin A, leucocin A, *Listeria*, growth, resistance, carbohydrate, bacteriocin

## Abstract

The aim of this study was to determine if different carbohydrates influence the growth of *Listeria monocytogenes* in the presence of carnocyclin A or leucocin A. *Carnobacterium maltaromaticum* ATCC PTA-5313 and *Leuconostoc gelidum* UAL187 were used to produce carnocyclin A and leucocin A, respectively. Growth curves were modeled for five strains of *L. monocytogenes* grown in basal medium supplemented with glucose, sucrose, fructose, mannose, or cellobiose, in the presence of carnocyclin A or leucocin A. The growth of *L. monocytogenes* to leucocin A or carnocyclin A was influenced by carbohydrate and/or strain. Carnocyclin A inhibited the growth of *L. monocytogenes* more than leucocin A. Growth in media containing glucose, mannose, and fructose increased the sensitivity of some strains of *L. monocytogenes* to bacteriocins, while growth in cellobiose and sucrose increased the resistance of *L. monocytogenes* to bacteriocins, as evidenced by a shorter lag phase. Strains of *L. monocytogenes* developed resistance to both leucocin A and carnocyclin A, but the time to develop resistance was longer when strains are treated with carnocyclin A. Carbohydrate influences the development of resistance of *L. monocytogenes* to the bacteriocins, but the ability of strains to develop resistance to leucocin A or carnocyclin A differs. Results of this study indicate that carbohydrates influence the ability of *L. monocytogenes* to grow in the presence of bacteriocins.

## Introduction

*Listeria monocytogenes* is a foodborne pathogen that causes infections with a fatality rate of 20–30% (Farber and Peterkin, 1991; Thomas et al., 2013). *L. monocytogenes* poses a food safety risk in relation to ready-to-eat (RTE) products as *L. monocytogenes* grows over a wide range of temperatures, including refrigeration temperatures (Zhu et al., 2005; Health Canada, 2011). According to the Centers for Disease Control and Prevention, the case frequency of listeriosis remained unchanged over the past 3 years (Centre for Disease Control, 2014) even though strict guidelines have been developed to control *L. monocytogenes* in RTE products (FSIS, 2014). With consumer demands for the food industry to increase availability of fresh, RTE and minimally processed products, novel methods of bio-preservation, such as bacteriocins, are needed. Bacteriocins are ribosomally synthesized antimicrobial peptides that inhibit the growth of foodborne pathogens and are an attractive alternative to traditional preservatives to improve food safety (Perez et al., 2014). However, *L. monocytogenes* can develop resistance to class IIa bacteriocins (Ramnath et al., 2000; Gravesen et al., 2002; Vadyvaloo et al., 2002, 2004a; Kaur et al., 2011), but little is known about resistance to circular bacteriocins.

Class IIa bacteriocins are small, hydrophobic, heat stable peptides that undergo minimal post-translational modification, and are degraded by various proteases (Cotter et al., 2005; Alvarez-Sieiro et al., 2016). Their mode of action is based on pore formation and disruption of ion gradients of the target cell (Héchard and Sahl, 2002). Previous work in our lab demonstrated that the five strains of *L. monocytogenes* that are recommended for use in challenge studies (Fugett et al., 2006) are resistant to 3.3 mM leucocin A in broth and will grow on vacuum packaged wieners that had 0.01 mM/cm^2^ leucocin A on the surface (Balay et al., 2017).

Resistance to class IIa bacteriocins has been linked to the mannose phosphotransferase system (Man-PTS; Dalet et al., 2001; Diep et al., 2007) and changes in cell membrane properties. Changes in the membrane can block pore formation by bacteriocins (Ramnath et al., 2004) due to increased membrane fluidity and a more positively charged cell wall (Vadyvaloo et al., 2002, 2004a,b). Resistance has also been correlated with the down regulation of the genes associated with the Man-PTS, which effects the transcription of enzyme II subunits (Gravesen et al., 2002). The membrane bound subunits are thought to provide a docking station for bacteriocins (Kjos et al., 2010, 2011). There are many sugar-PTS that are dedicated to specific carbohydrates that are transported across the membrane. It may be possible that a culture does not become resistant to a bacteriocin if the specific PTS involved in bacteriocin docking is required for carbohydrate transport to support growth. However, a study by Tessema et al. (2009), showed spontaneous mutants of *L. monocytogenes* that were highly-resistant (IC_50_, >104 ng/ml) to sakacin P were able to grow significantly faster in media supplemented with mannose and glucose than low-level resistant (IC_50_, <104 ng/ml) spontaneous mutant strains of *L. monocytogenes*. Additionally, there was upregulation of the *mptA* in some stains of *L. monocytogenes* that were highly-resistant to Class IIa bacteriocins compared to the sensitive, wild type strains (Gravesen et al., 2004; Tessema et al., 2009), which indicates there could be additional mechanisms for cells to form resistance to bacteriocins that are not fully understood yet.

Leucocin A produced by *Leuconostoc gelidum* UAL187 is a 37-residue class IIa bacteriocin with a molecular mass of 3,903 Da, which docks with the Man-PTS (Hastings and Stiles, 1991; Drider et al., 2006). Carnocyclin A, a 60-residue circular bacteriocin with potent antilisterial activity, is produced by *Carnobacterium maltaromaticum* ATCC PTA-5313 (Martin-Visscher et al., 2008; van Belkum et al., 2011). Circular bacteriocins have some distinguishing features including an amide bond that connects the N- and C- termini, and increased stability against proteases (van Belkum et al., 2011). Carnocyclin A forms anion selective channels in the lipid membrane, therefore carnocyclin A does not require a docking molecule to form a pore (Gong et al., 2009). However, more research is needed to fully understand if ion selective pores are the only mode of action for carnocyclin A (Drider et al., 2006; Gong et al., 2009). As cyclic bacteriocins interact differently with the membrane compared to Class IIa bacteriocins, there may be different mechanisms for bacteriocin resistance in *L. monocytogenes*.

The objective of this research was to determine the effect of different carbohydrates on the growth kinetics of *L. monocytogenes* strains associated with food borne illness in the presence of two bacteriocins that differ in class and mode of action, leucocin A and carnocyclin A.

## Materials and Methods

### Bacterial Strains and Preparation

Outbreak strains of *L. monocytogenes* (FSL N1-227, FSL R2-499, FSL N3-013, FSL J1-177, FSL C1-056) developed for use in challenge studies were obtained from Cornell University (Fugett et al., 2006). *C. maltaromaticum* ATCC-PTA 5313, carnocyclin A producer, and *Leuconostoc gelidum* UAL187, leucocin A producer, were obtained from the University of Alberta Lactic Acid Bacteria culture collection. All strains were stored in 30% glycerol in media at -80°C until needed.

Prior to use in experiments, cultures were streaked onto All Purpose Tween agar (APT; Becton, Dickinson and Company, Franklin Lakes, NJ, United States), and a single colony was inoculated into broth media. *C. maltaromaticum* ATCC-PTA 5313 and *Leuconostoc gelidum* UAL187 were grown anaerobically at 25°C in APT broth and semi-defined Casamino Acids (CAA) broth without Tween^®^ 80 (Hastings et al., 1991; Balay et al., 2017), respectively. Strains of *L. monocytogenes* were grown at 32°C in APT broth.

### Production and Purification of Bacteriocins

Leucocin A was purified using a protocol described in Balay et al. (2017). Carnocyclin A was purified using a modified protocol of Martin-Visscher et al. (2008). *C. maltaromaticum* ATCC PTA-5313 was grown in 1 L APT broth at 25°C for 24 h. For purification, the culture, including the cells, was loaded onto an Amberlite^®^ XAD-16N column (100 g/L; 2.5 cm × 50 cm; 5 ml/min; Sigma-Aldrich^®^, St. Louis, MO, United States) at 6°C. Resin was pre-conditioned with 100% isopropanol (IPA) and rinsed with sterile distilled water (dH_2_O). The column was washed sequentially at a flow rate of 10 ml/min with 500 ml of dH_2_O, 500 ml of 30% (v/v) ethanol (EtOH), 500 ml of 40% (v/v) IPA, 500 ml 70% (v/v) IPA, and carnocyclin A was eluted with 500 ml 70% (v/v) IPA, pH 2. The fraction was concentrated using a Buchi^®^ rotary evaporator (Brinkman Instruments, Westbury, NY, United States) at 30°C under vacuum to 10 ml. The concentrated fraction was loaded onto a Sep-Pak^®^ Vac 12cc (2 g) C18 cartridge (Waters, Milford, MA, United States), which had been pre-washed with 50 ml methanol followed by 50 ml dH_2_O. The column was washed consecutively with 50 ml of dH_2_O, 50 ml of 30% (v/v) EtOH, 50 ml of 30% (v/v) acetonitrile (ACN), 50 ml 40% (v/v) IPA, 50 ml 70% (v/v) IPA, and carnocyclin A was eluted with 100 ml 70% (v/v) IPA, pH 2. This fraction was concentrated to 2 ml using a rotary evaporator and lyophilized (Labconco^®^ FreezeZone, Kansas City, MO, United States). The lyophilized sample was suspended in 5 ml of dH_2_O for reverse-phase high performance liquid chromatography (RP-HPLC; Beckman System Gold, Beckman Coulter, Inc., Mississauga, ON, Canada). Samples of 1 ml were injected onto a C8 column (5 μm particle size, 10 mm × 250 mm, Vydac 208TP510, Hichrom, Berkshire, United Kingdom). The RP-HPLC mobile phase consisted of (A) IPA and (B) H_2_O, each containing 0.1% trifluoroacetic acid. A gradient RP-HPLC method was used, consisting of a 8 min hold at 20% A; 30 min increase of A from 20 to 86%; 5 min hold at 86% A; and a 3 min decrease from 86 to 20% A, with a flow rate of 1 ml/min and ultraviolet detection set at 220 nm. Carnocyclin A eluted as a sharp peak (retention time 23 to 24 min). After purification, all carnocyclin A fractions were combined, lyophilized and stored at -20°C until needed. For experimental use, carnocyclin A was suspended in dH_2_O to the desired concentration.

The purified samples were subjected to matrix-assisted laser desorption ionization-time of flight mass spectrometry (MALDI-TOF) to confirm the presence of leucocin A or carnocyclin A.

### Growth Kinetics of *L. monocytogenes* Grown in Different Carbohydrates Treated With Leucocin A or Carnocyclin A

For growth experiments, basal media supplemented with individual carbohydrates was used. For 1 L of basal medium the following formulation was used: 10 g Proteose Peptone No. 3 (Becton, Dickinson and Company), 10 g Beef Extract (Becton, Dickinson and Company), 5 g Yeast Extract (Becton, Dickinson and Company), 1 g Tween^®^ 80, 2 g disodium phosphate, 1 ml sterile basal media salt solution [10 g magnesium sulfate, 5 g manganese sulfate dissolved in 100 ml dH_2_O], and for each liter of basal medium, 20 g of a carbohydrate (glucose, sucrose, fructose, mannose, or cellobiose) was added.

To determine growth kinetics, five series of four wells in separate 96 well microtiter plates(s) for each strain were filled with 50 μl of each of the five basal media supplemented with individual carbohydrates. An additional 100 μl of each basal media was added to the first well of each series as a negative control. For each microtiter plate, an overnight culture of one strain of *L. monocytogenes* was centrifuged, suspended in sterile 0.1% (w/v) peptone water (Becton, Dickinson and Company) and serially diluted with peptone water with the final dilution made in basal media. To the second well of each series, 50 μl of basal media and 50 μl of one strain of *L. monocytogenes* was added. The third series of wells contained basal media, *L. monocytogenes* and leucocin A; and the fourth series included *L. monocytogenes* and carnocyclin A. The final concentration of bacteriocins was 3.3 mM and cell density was ∼10^4^ CFU per well, confirmed by plate count on APT agar. The concentration of bacteriocin was chosen as it is equal or higher than the MIC against *L. monocytogenes* determined on a critical dilution assay on agar after incubation for 24 h (data not shown; Balay et al., 2017) but did not prevent growth of resistant derivatives upon prolonged incubation. Preliminary experiments indicated that the response of the five strains of *L. monocytogenes* to challenge with bacteriocins was similar when pre-cultures were prepared with APT (glucose) or supplemented basal media containing carbohydrates other than glucose.

Each microtiter plates was covered with a clear optical adhesive film (MicroAmp, Applied Biosystems, Thermo Fischer Scientific, Burlington, ON, Canada) to prevent evaporation, loss of culture or contamination and placed in a spectrophotometer (Multiskan Ascent, Thermo Electron Corporation, Shanghai, China) and incubated at 25°C for 72 h. Each plate was shaken every 2 h for 10 s at 700 RPM, and the optical density (630 nm) of each well was recorded. Growth curves were generated using Mulitskan Ascent Software (Version 2.6, Thermo Fischer Scientific). The experiment was replicated independently three times.

### Identify Phenotypic Differences of the Subpopulation to Bacteriocins

To characterize phenotypical differences of the subpopulation that grew in the presence of either bacteriocin from the growth experiments (see section “Growth Kinetics of *L. monocytogenes* Grown in Different Carbohydrates Treated with Leucocin A or Carnocyclin A”), two strains were selected for further testing; FSL R2-499 and FSL C1-056. Both strains of *L. monocytogenes* were grown in basal media supplemented with either mannose or cellobiose and treated with 3.3 mM leucocin A or carnocyclin A.

To compare growth kinetics of the original cultures compared to that each subpopulation first a growth experiment under similar conditions as outlined in Section “Growth Kinetics of *L. monocytogenes* Grown in Different Carbohydrates Treated with Leucocin A or Carnocyclin A,” followed by isolation of individual colonies from the subpopulation that grew, which then were used in a second growth experiment with the same conditions as the first growth experiment.

To determine the growth kinetics for the original cultures FSL R2-499 and FSL C1-05, two series (one for each carbohydrate) of four wells was used in separate 96 well microtiter plates(s) for each strain. The total well volume used was 200 μl. The first well of each series was filled with 200 μl of each basal media supplemented with individual carbohydrates (mannose or cellobiose) as a negative control. For each microtiter plate, an overnight culture of one strain of *L. monocytogenes* was centrifuged, suspended in sterile 0.1% (w/v) peptone water (Becton, Dickinson and Company) and serially diluted with peptone water with the final dilution made in basal media. To the second well of each series, 150 μl of basal media and 50 μl of one strain of *L. monocytogenes* was added. The third series of wells contained basal media, *L. monocytogenes* and leucocin A; and the fourth series included *L. monocytogenes* and carnocyclin A. The final concentration of bacteriocins was 3.3 mM and cell density was ∼10^6^ CFU per well, confirmed by plate count on APT agar. Each microtiter plates was covered with a clear optical adhesive film (MicroAmp, Applied Biosystems, Thermo Fischer Scientific, Burlington, ON, Canada) to prevent evaporation, loss of culture or contamination and placed in a spectrophotometer (Multiskan Ascent, Thermo Electron Corporation, Shanghai, China) and incubated at 25°C for 40 h. Each plate was shaken every 2 h for 10 s at 700 RPM, and the optical density (630 nm) of each well was recorded. Growth curves were generated using Mulitskan Ascent Software (Version 2.6, Thermo Fischer Scientific).

Following the first growth experiment seals were removed from microtiter plates and a loopful of culture was obtained from each well of the both series. The culture was then streaked on APT agar to obtain single colonies. Plates were incubated at 32°C for 24 h before individual colonies were picked and cultured into APT broth at 32°C for 24 h. The same growth experiment then followed for the isolated subpopulations as outlined above with the original cultures. The experiment was replicated independently four times.

### Predictive Modeling of Growth Curves

Growth curves were fitted to a modified logistic model (Zwietering et al., 1990) to assess how maximal growth rates (µ) and lag phase (λ) were affected by the presence of leucocin A or carnocyclin A when *L. monocytogenes* was grown in different carbohydrates. SigmaPlot 12.5 was used for all curve fit procedures.

### Statistical Analysis

Data were analyzed using the PROC ANOVA of SAS Studio (Release 3.5 University Edition, SAS Institute, Inc., Cary, NC, United States) with strain, carbohydrate and bacteriocins as categorical variables and lag phase and growth rate as dependent variables. To determine significant differences among means, Tukey test with a 95% confidence interval was used to determine significant interactions. To determine the specific impact of the main effects of strain, carbohydrate or bacteriocin, one-way ANOVA was done with differences among means determined with a Tukey test.

## Results

### Purification

The novel protocol for purification of leucocin A and carnocyclin A recovered 2.3 and 4.8 mg/L of leucocin A and carnocyclin A, respectively. Purification yields of leucocin A and carnocyclin A were 5-and 2.5-fold higher, respectively, than the yields reported by Hastings et al. (1991) and Martin-Visscher et al. (2008). The molecular mass of the purified leucocin A and carnocyclin A was 3,930.03 and 5,863.50 Da, respectively, matching prior reports (Hastings et al., 1991; Martin-Visscher et al., 2008).

### Predictive Modeled Growth Curves

**FIGURE 1:**
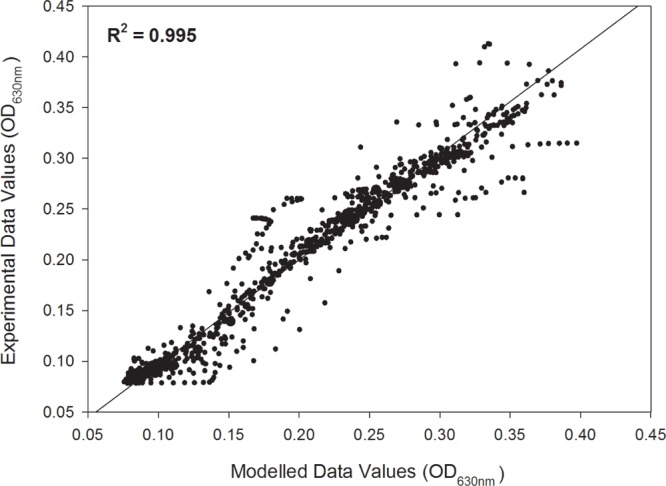
Unity plot of experimental optical density data values versus the fitted (modeled) data values of all five *Listeria monocytogenes* (FSL N1-227, FSL R2-499, FSL N3-013, FSL J1-177, FSL C1-056) grown in each of the carbohydrates (glucose, sucrose, fructose, mannose, cellobiose) for the control, or treated with 3.3 mM leucocin A or carnocyclin A (*n* = 3).

**FIGURE 2:**
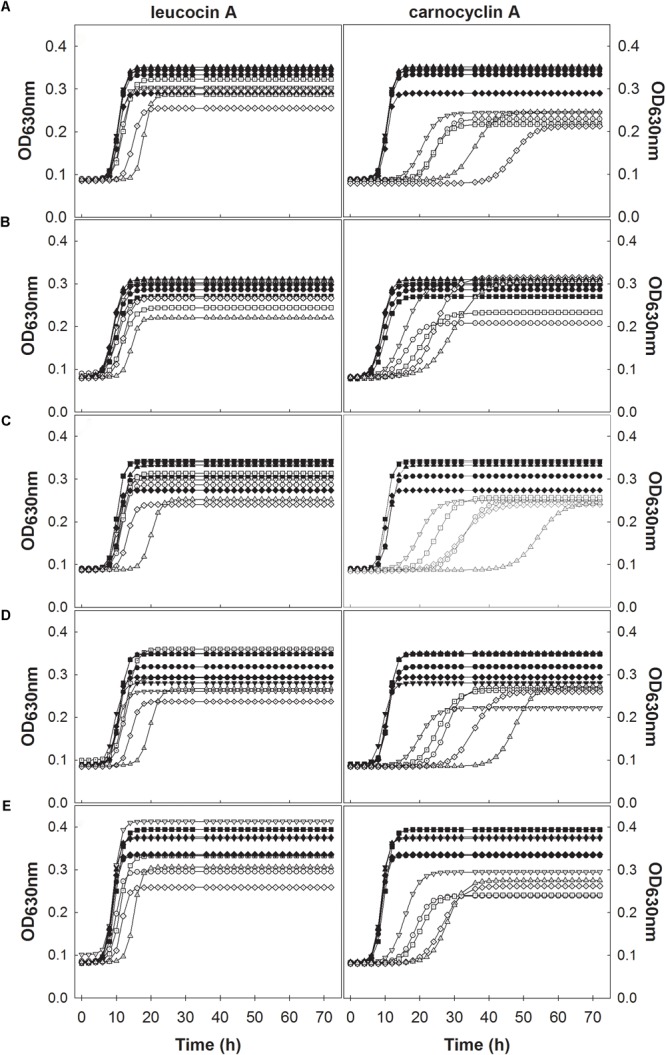
Modeled growth curves of *L. monocytogenes* FSL N1-227 (●,○); FSL R2-499 (▼,▽); FSL N3-013 (■,□), FSL J1-177 (♦,♢), FSL C1-056 (▲,△), with the addition of bacteriocins (open symbols) or without bacteriocin (closed symbols) in media supplemented with different carbohydrates glucose **(A)**, sucrose **(B)**, fructose **(C)**, mannose **(D)**, cellobiose **(E)** and measured at 630 nm every 2 h for 72 h at 25°C. Plots in the left column were treated with leucocin A and those in the right column were treated with carnocyclin A (*n* = 3).

Individual growth curves were fitted to a modified logistic model with an *R*^2^ ≥ 0.99 with an overall fit of *R*^2^= 0.995 as shown in the unity plot (**Figure 1**). Each of the experimental growth curves with the modeled curve for each growth condition is shown in Supplementary Figures S1–S7. *L. monocytogenes* FSL N1-227 grown in fructose with carnocyclin A and FSL J1-177 grown in glucose with carnocyclin A gave a growth curve that did not precisely fit the sigmoidal shape of the logistic model (Supplementary Figures S1, S4), but the *R*^2^ was > 0.99. The modeled growth curves for each strain of *L. monocytogenes* grown in the presence of each carbohydrate and leucocin A or carnocyclin A are shown in **Figure 2**. Leucocin A at a concentration of 3.3 mM did not inhibit the growth of any of the strains of *L. monocytogenes* in the presence of any of the tested carbohydrates (**Figure 2A**), demonstrating that all strains developed resistance against leucocin A. Only two of the five strains shows an extended lag phase in presence of leucocin A in presence of some but not all carbohydrates (**Table 1**), indicating that the development of resistance is depended on the strain and the carbohydrate source. Carnocyclin A extended the lag phase and reduced the growth rate of all strains; the extent of inhibition again depended on individual strains and/or carbohydrates (**Figure 2B**).

**Table 1:** Least squared means of maximal growth rate and lag phase of *L. monocytogenes* determined at 25°C in basal medium supplemented with different carbohydrates in the presence of leucocin A or carnocyclin A (*n* = 3).

		Control		Leucocin A		Carnocyclin A	
		µ^∗^	λ^†^	µ	λ	µ	λ
**FSL N1-227**	Glucose	0.31 ± 0.03^a^	7.69 ± 0.92^b^	0.26 ± 0.02^bxy^	8.52 ± 1.35^by^	0.12 ± 0.01^c^	19.56 ± 6.49^a^
	Sucrose	0.20 ± 0.01^ay^	5.47 ± 0.88^b^	0.17 ± 0.01^ay^	6.55 ± 0.60^aby^	0.10 ± 0.01^b^	10.42 ± 2.79^ayz^
	Fructose	0.31 ± 0.02^ay^	8.55 ± 0.97^b^	0.26 ± 0.01^bx^	8.73 ± 1.15^by^	0.10 ± 0.01^cxy^	25.64 ± 2.45^ay^
	Mannose	0.28 ± 0.03^ay^	7.79 ± 0.85^b^	0.22 ± 0.01^axy^	8.72 ± 1.30^by^	0.13 ± 0.05^b^	22.13 ± 6.88^axy^
	Cellobiose	0.36 ± 0.01^a^	6.38 ± 0.59^b^	0.30 ± 0.01^bxy^	7.22 ± 0.86^by^	0.13 ± 0.01^cxy^	13.55 ± 2.40^axy^
**FSL R2-499**	Glucose	0.33 ± 0.02^a^	7.60 ± 0.26^b^	0.29 ± 0.03^ax^	7.91 ± 0.64^by^	0.12 ± 0.03^b^	14.99 ± 4.35^a^
	Sucrose	0.25 ± 0.01^ax^	5.60 ± 0.17	0.22 ± 0.01^ax^	6.12 ± 0.41^y^	0.13 ± 0.02^b^	8.76 ± 2.47^z^
	Fructose	0.32 ± 0.02^axy^	7.41 ± 0.43^b^	0.27 ± 0.02^ax^	7.52 ± 0.81^by^	0.11 ± 0.01^bx^	13.61 ± 2.54^ay^
	Mannose	0.24 ± 0.11^ay^	6.26 ± 2.77^b^	0.24 ± 0.10^bx^	7.69 ± 2.88^by^	0.11 ± 0.04^c^	14.07 ± 5.55^ay^
	Cellobiose	0.38 ± 0.03^a^	6.18 ± 0.15^b^	0.34 ± 0.02^ax^	6.52 ± 0.19^by^	0.15 ± 0.01^bx^	10.13 ± 0.64^ay^
**FSL N3-013**	Glucose	0.33 ± 0.00^a^	7.83 ± 0.46^b^	0.24 ± 0.02^bxy^	8.44 ± 0.69^by^	0.10 ± 0.02^c^	18.18 ± 5.94^a^
	Sucrose	0.20 ± 0.01^ay^	6.13 ± 0.28^b^	0.15 ± 0.00^bz^	7.11 ± 0.53^by^	0.09 ± 0.00^c^	12.60 ± 2.50^axyz^
	Fructose	0.33 ± 0.00^axy^	7.46 ± 0.50^b^	0.23 ± 0.02^bxy^	8.07 ± 0.82^by^	0.10 ± 0.01^cxy^	18.13 ± 5.01^ay^
	Mannose	0.31 ± 0.02^axy^	8.07 ± 0.36^b^	0.21 ± 0.02^bxy^	8.77 ± 0.58^by^	0.11 ± 0.02^c^	18.45 ± 4.99^ay^
	Cellobiose	0.34 ± 0.03^a^	6.61 ± 0.29^b^	0.27 ± 0.02^by^	7.61 ± 0.52^by^	0.11 ± 0.01^cxy^	14.17 ± 3.41^axy^
**FSL J1-177**	Glucose	0.34 ± 0.02^a^	8.17 ± 0.65^b^	0.22 ± 0.02^by^	11.54 ± 2.52^abxy^	0.09 ± 0.01^c^	39.89 ± 21.17^a^
	Sucrose	0.25 ± 0.01^ax^	5.42 ± 0.40^b^	0.23 ± 0.01^ax^	8.60 ± 1.53^bxy^	0.12 ± 0.05^b^	17.05 ± 4.58^axy^
	Fructose	0.36 ± 0.01^ax^	7.86 ± 0.73^b^	0.22 ± 0.04^bxy^	10.31 ± 1.93^axy^	0.07 ± 0.01^cy^	22.51 ± 9.83^ay^
	Mannose	0.36 ± 0.02^ax^	7.97 ± 0.79	0.22 ± 0.02^bxy^	11.19 ± 2.61^y^	0.09 ± 0.02^c^	27.87 ± 14.51^xy^
	Cellobiose	0.39 ± 0.01^a^	6.29 ± 0.37^b^	0.30 ± 0.02^by^	9.12 ± 1.77^abxy^	0.10 ± 0.03^cy^	19.19 ± 7.04^axy^
**FSL C1-056**	Glucose	0.33 ± 0.02^a^	7.87 ± 0.88^b^	0.29 ± 0.00^bx^	15.27 ± 1.70^bx^	0.09 ± 0.01^c^	28.82 ± 6.94^a^
	Sucrose	0.25 ± 0.02^ax^	5.50 ± 1.07^c^	0.22 ± 0.00^bx^	11.43 ± 1.99^bx^	0.08 ± 0.01^c^	19.43 ± 2.21^ax^
	Fructose	0.29 ± 0.02^ay^	8.45 ± 1.45^b^	0.19 ± 0.01^by^	16.57 ± 2.57^bx^	0.07 ± 0.00^cy^	45.54 ± 9.33^ax^
	Mannose	0.32 ± 0.02^axy^	8.09 ± 1.04^c^	0.19 ± 0.01^by^	16.15 ± 2.16^bx^	0.10 ± 0.01^c^	40.39 ± 4.93^ax^
	Cellobiose	0.36 ± 0.02^a^	6.14 ± 0.91^b^	0.29 ± 0.01^by^	12.09 ± 1.75^bx^	0.13 ± 0.01^cxy^	21.83 ± 4.28^ax^

*^∗^Maximal growth rate (h^-*1*^), ^†^lag phase (h); ^*a,b,c*^across rows indicate significantly different (*P* ≤ 0.05) μ_*m*_ or λ values based on the presence (or lack thereof) of bacteriocin for each strain of *L. monocytogenes* grown in each different carbohydrates; ^*x,y,z*^down columns indicate significantly different (*P* ≤ 0.05) μ_*m*_ or λ values among strains grown in the same carbohydrate for each bacteriocin and the control.*

### Growth Rate as a Function of Lag Phase

**FIGURE 3:**
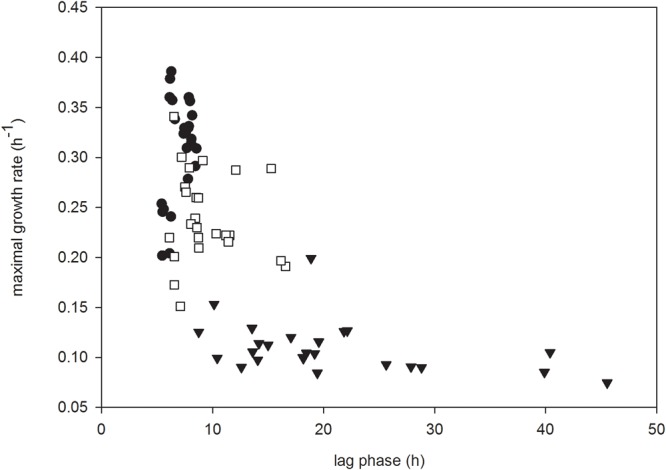
Growth rate plotted as a function of lag phase for all strains of *L. monocytogenes* grown in all carbohydrates for the control (●) or in the presence of leucocin A (□) or carnocyclin A (▼) (*n* = 3).

Growth rate was plotted as a function of lag phase duration for each strain grown in each carbohydrate based on bacteriocin treatment to assess trends in the data (**Figure 3**). In the absence of either bacteriocin, the lag phases of all strains of *L. monocytogenes* were of similar duration, although there was greater variation among their subsequent growth rates (**Figure 3**). In the presence of carnocyclin A this situation was reversed, in that the lag phase of *L. monocytogenes* varied considerably, but their subsequent growth rates were broadly similar. In the presence of leucocin A there was less variation among lag phases, and among subsequent growth rates, than in the absence of bacteriocin, or in the presence of carnocyclin A (**Figure 3**).

### Impact of Carbohydrate on Growth of *L. monocytogenes*

**FIGURE 4:**
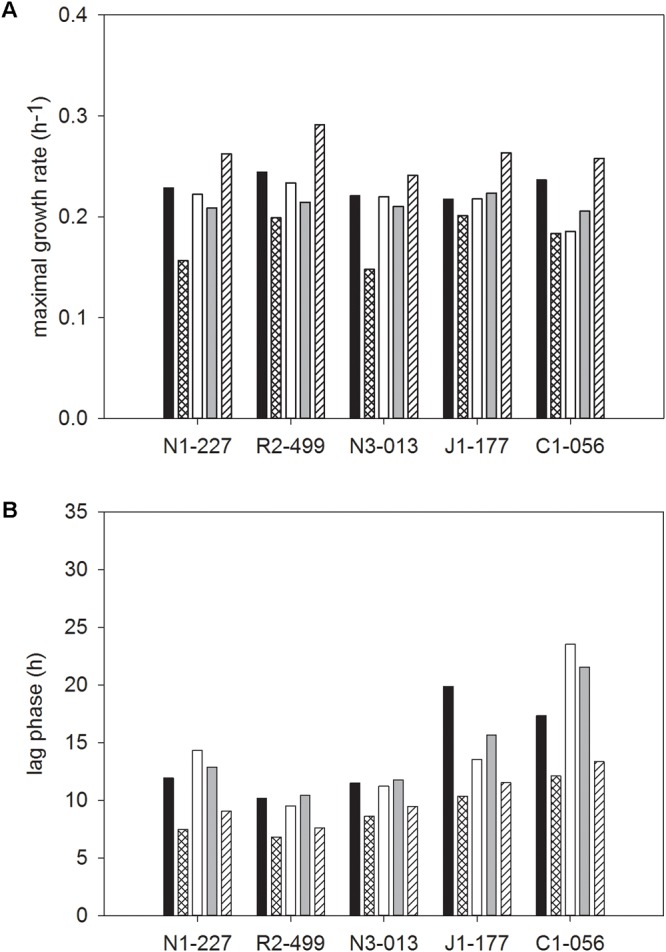
Least squared means of significant two-way interactions for the maximal growth rate **(A)** and lag phase **(B)** of each strain of *L. monocytogenes* (FSL N1-227, FSL R2-499, FSL N3-013, FSL J1-177, FSL C1-056) grown in glucose (■), sucrose (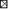), fructose (□), mannose (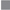), cellobiose (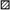), regardless of bacteriocin treatment (*n* = 3).

The two-way interaction means for the growth rate and lag phase of cultures grown in media with individual carbohydrates, across all bacteriocin treatments are shown in **Figure 4**. There were significant interactions between strain and carbohydrate that resulted in differences in growth rate and lag phase for individual carbohydrates (**Figures 4A,B**). For FSL N1-227 and FSL N3-013, the maximal growth rate was significantly lower when strains were grown in sucrose, whereas growth in cellobiose resulted in a significantly higher growth rate for all strains (**Figure 4A**). The lag phases of *L. monocytogenes* FSL C1-056 and FSL J1-177 were significantly longer when grown in mannose, glucose, and fructose (**Figure 4B**).

### Impact of Bacteriocin and Carbohydrate on the Growth of *L. monocytogenes*

**FIGURE 5:**
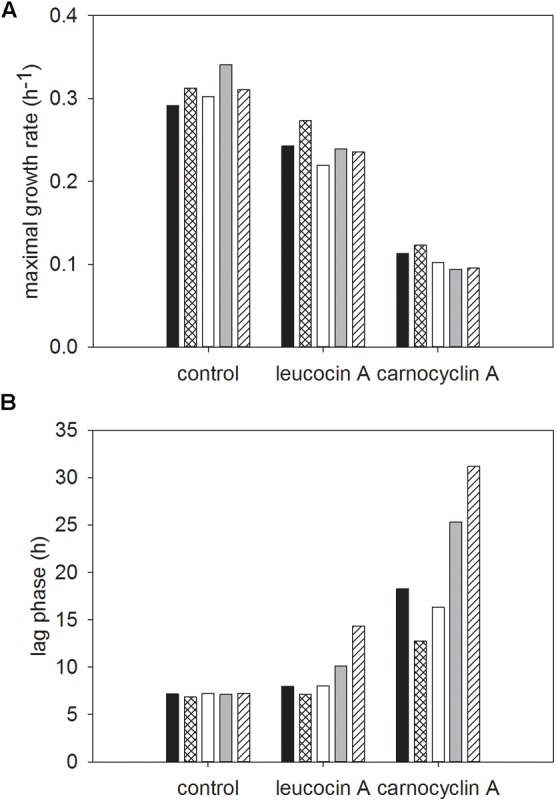
Least squared means of significant two-way interactions for the maximal growth rate **(A)** and lag phase **(B)** of each strain of *L. monocytogenes* FSL N1-227 (■), FSL R2-499 (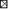), FSL N3-013 (□), FSL J1-177 (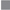), FSL C1-056 (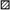) by control or bacteriocin treatment (leucocin A, carnocyclin A), regardless of carbohydrate (*n* = 3).

**FIGURE 6:**
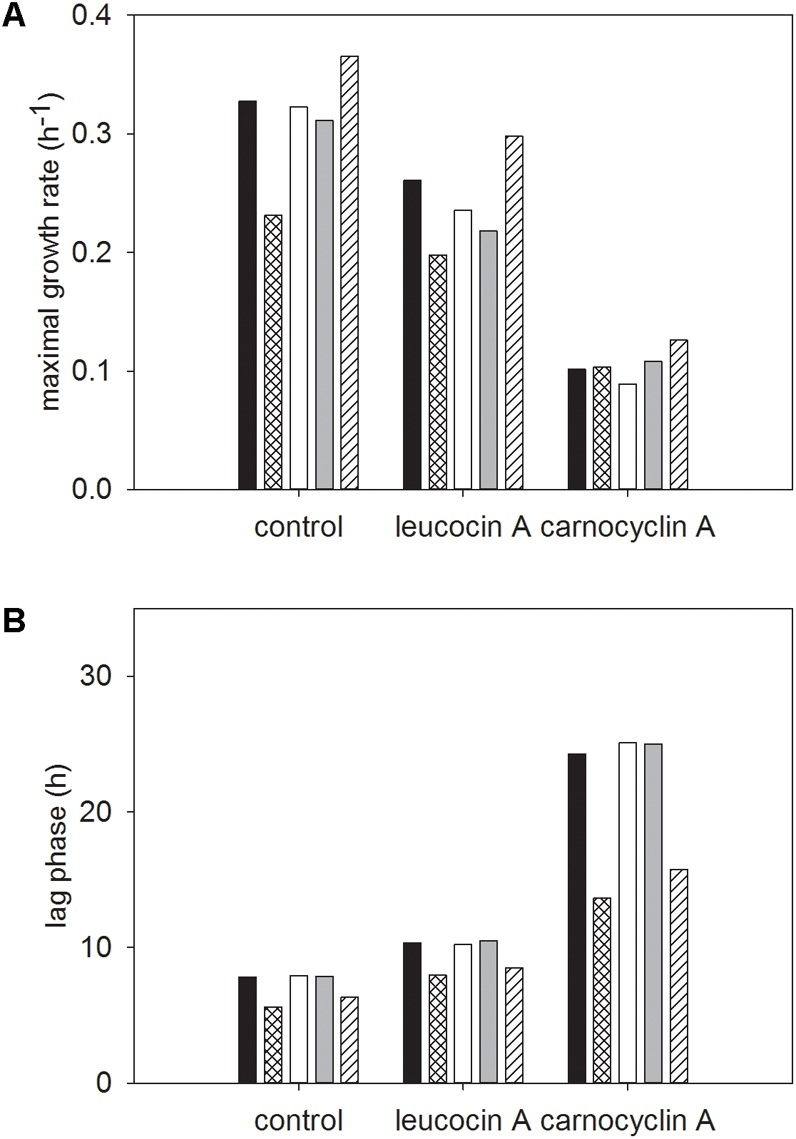
Least squared means of significant two-way interactions for the maximal growth rate **(A)** and lag phase **(B)** of each carbohydrate glucose (■), sucrose (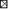), fructose (□), mannose (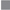), cellobiose (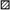) by control or bacteriocin treatment (leucocin A, carnocyclin A), regardless of strain of *L. monocytogenes* (*n* = 3).

There were significant two-way interactions between strain and bacteriocin treatment, and carbohydrate and bacteriocin treatment (**Figures 5, 6**, respectively).

For each strain, regardless of carbohydrate, leucocin A resulted in a significantly lower growth rate than the untreated control, while carnocyclin A resulted in a significantly lower growth rate than strains treated with leucocin A or the untreated control (**Figure 5A**). When treated with leucocin A, FSL R2-499 had the fastest growth rate (*P* < 0.05) and FSL N3-013 had the slowest growth rate (*P* < 0.05; **Figure 5A**). All strains had the same lag phase when not treated with bacteriocin, but treatment of strains FSL C1-056 and FSL J1-177 with leucocin A resulted in a significantly longer lag phase than that observed for the untreated control (**Figure 5B**). Treatment with carnocyclin A significantly increased the lag phase for all cultures compared to the untreated control (**Figure 5B**). The lag phase of strains FSL N1-227, FSL C1-056, and FSL J1-177 were significantly longer when treated with carnocyclin A compared to those treated with leucocin A.

Regardless of strain, growth in cellobiose resulted in the highest growth rate (*P* < 0.05) within each bacteriocin treatment (**Figure 6A**). Growth in media with carnocyclin A and any of the carbohydrates significantly reduced growth rates (**Figure 6A**). Sucrose resulted in the slowest growth rate for the untreated control and cells treated with leucocin A, whereas fructose resulted in the slowest growth rate for all strains treated with carnocyclin A (**Figure 6A**). Compared to the untreated control, there was no difference (*P* > 0.05) in lag phase of cultures treated with leucocin A regardless the carbohydrate, but the lag phase increased (*P* < 0.05) when cultures were treated with carnocyclin A (**Figure 6B**). Growth in glucose, fructose, or mannose increased (*P* < 0.05) the lag phase in media with carnocyclin A compared to cultures grown in the presence of leucocin A (**Figure 6B**).

### Impact of Individual Factors on Growth Rate and Lag Phase of *L. monocytogenes*

The impact of strain, bacteriocin or carbohydrate was determined by one-way ANOVA (**Table 1**). Strain did not affect growth rate when cultures were grown in glucose or cellobiose without bacteriocin (**Table 1**). Growth rate was significantly reduced for strain FSL N1-227 when grown in sucrose, fructose, and mannose compared to the untreated control group (**Table 1**). Strain had no effect on the lag phase for the untreated control group regardless of the carbohydrate (**Table 1**).

Leucocin A significantly decreased the growth rates of all strains when grown in glucose, fructose, or cellobiose, except for FSL R2-499 (**Table 1**). Strain FSL R2-499 was not affected by leucocin A except when grown in mannose, which significantly decreased the growth rate compared to the untreated control (**Table 1**). When FSL N1-227 was grown in mannose it was resistant to leucocin A as there was no effect on growth rate compared to the untreated control, which was the exact opposite for FSL R2-499 (**Table 1**). Strain FSL C1-056 had the slowest growth rate when grown in fructose, mannose, or cellobiose, and treated with leucocin A (**Table 1**). However, when FSL C1-056 was grown in glucose or sucrose it was one of the fastest growing strains despite having a significantly increased lag phase compared to the untreated control in sucrose and mannose (**Table 1**). In all carbohydrates, when FSL C1-056 was treated with leucocin A there was a significant increase in lag phase (**Table 1**).

Carnocyclin A significantly decreased the growth rates of all strains compared to the untreated control, but fructose and cellobiose caused variation in growth rates among strains (**Table 1**). Carnocyclin A significantly increased the lag phase for all strains when grown in glucose, fructose, or cellobiose compared to the untreated control. There was no difference in lag phase across strains when grown in glucose and treated with carnocyclin A. When grown in sucrose FSL R2-499 had increased resistance to carnocyclin A as the lag phase was not affected by bacteriocin treatment. Strain FSL C1-056 had an increase in lag phase when grown in sucrose in the presence of carnocyclin A compared to strains FSL N1-227 and FSL R2-499. Similarly, FSL C1-056 had the longest lag phase compared to all other strains when grown in fructose and treated with carnocyclin A. Strain FSL C1-056 had significantly longer lag phase when treated with carnocyclin A and grown in mannose when compared to strains FSL R2-499and FSL N3-013 (**Table 1**).

### Phenotypical Characteristics of the Growth Kinetics of the Subpopulation

The growth kinetics of cultures was analyzed after growth in the presence of both bacteriocins to compare the maximal growth rate and lag phase to that of original cultures.

**FIGURE 7:**
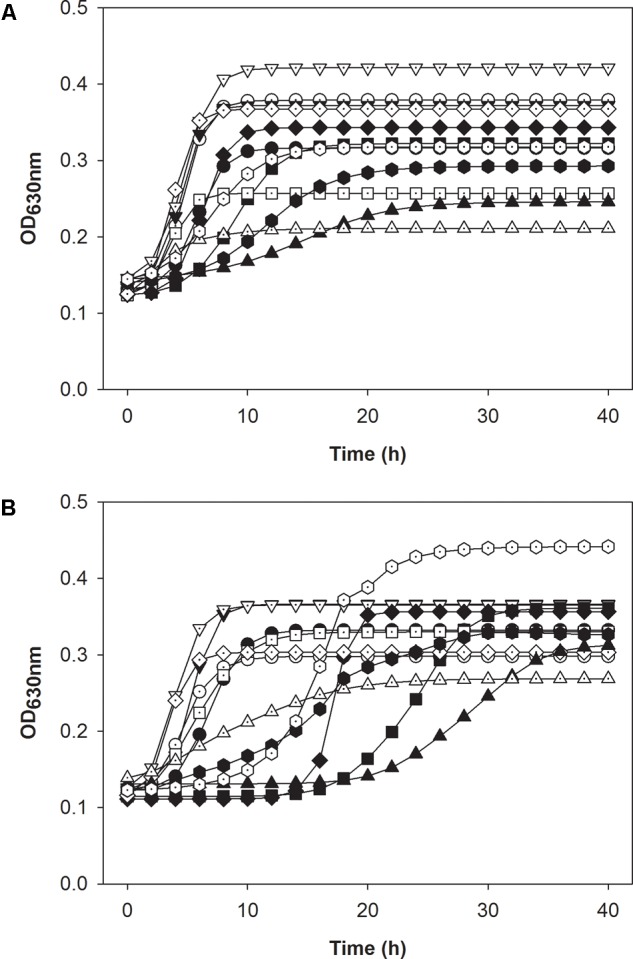
Modeled growth curve data of *L. monocytogenes* strains FSL R2-499 **(A)** and FSL C1-056 **(B)**. Both panels compare the growth of after inoculation with adapted pre-cultures that grown in presence of 3.3 mM bacteriocins (open symbols) to the growth of after inoculation with “naïve” pre-cultures that were not exposed to bacteriocins (closed symbols). Cultures were grown in presence of mannose (●,○); mannose and leucocin A (■,□); mannose and carnocyclin A (▲,△); cellobiose (▼,▽); cellobiose and leucocin A (♦,♢); cellobiose and carnocyclin A (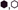) at 25 °C for 40 h. Optical density was measured at 630 nm (*n* = 4).

The original cultures prepared in absence of bacteriocin grew in both sugars (**Figures 7A,B** and Supplementary Table S1); growth parameters were slightly modified, which reflects the different culture conditions for the inoculum of the respective cultures. *L. monocytogenes* FSL C1-056 had longer lag phases in both carbohydrates when treated with either bacteriocin compared to FSL R2-499 (**Figure 7** and Supplementary Table S1). Carnocyclin A reduced the maximal growth rate for both strains grown in both carbohydrates compared to that of the control (**Figure 7** and Supplementary Table S1).

After pre-adaptation of *L. monocytogenes* to bacteriocins, neither carnocyclin A nor leucocin A increased the lag phase of the cultures (**Figure 7** and Supplementary Table S1). This indicates that adaptation to the bacteriocins eliminates or reduces the time for development of resistance upon sub-culture in presence of bacteriocins. One noticeable exception was *L. monocytogenes* FSL C1-056. The lag phase of this strain in mannose or mannose–carnocyclin A did not differ after pre-adaptation to carnocyclin A; however, the strain still showed an extended lag phase to carnocyclin A after adaptation in presence of cellobiose (Supplementary Table S1).

## Discussion

The application of bacteriocins in food to inhibit the growth of *L. monocytogenes* requires a better understanding of how carbohydrates affect the efficacy of bacteriocins and development of resistance.

Leucocin A decreased the growth rate of some strains of *L. monocytogenes* in most carbohydrates, but extend the lag phase only for few strains. When the growth of the *L. monocytogenes* is delayed due to the presence of leucocin A, development of resistance to the bacteriocin occurs allowing the growth of the culture after an initial reduction cell counts (Balay et al., 2017). The observation that leucocin A concentrations exceeding the MIC on agar-assays did not significantly alter growth parameters in liquid culture indicates that resistance develops in a relatively large proportion of the population, and that fitness cost of resistance are marginal. Results from this study also confirm that development of resistance to bacteriocins results in a stable phenotype. Bacteriocin resistance as stable phenotype is supported by the observation that bacteriocins increased the lag phase in the original cultures but not in cultures that were adapted to the respective bacteriocins.

Our results showed that growth in different carbohydrates supported development of resistance to bacteriocins, but the effect depended on the strain and the carbon source. For class IIa bacteriocins, concentrations exceeding the MIC 5- to 1,000-fold can induce resistance in *L. monocytogenes* (Duffes et al., 2000; Kaur et al., 2011). Although others have reported inhibition of *L. monocytogenes* in the presence of carnocyclin A (Gong et al., 2009; Liu et al., 2014), to the best of our knowledge this is the first report of resistance to carnocyclin A in strains of *L. monocytogenes*. In the current study, all of the strains used were able to grow in the presence of carnocyclin A, although the growth rate and lag phase were impacted by carbohydrate.

Mannose, glucose, and fructose reduced the growth rate for cells treated with leucocin A, with the exception of strain FSL R2-499. The increased sensitivity to leucocin A, as evidenced by the reduced growth rate, may relate to a requirement for use of the Man-PTS for carbohydrate uptake. This supports the hypothesis that growth of *L. monocytogenes* in presence of a specific carbohydrate increases the sensitivity of *Listeria* to the bacteriocin if the sugar transport system is also used as a bacteriocin docking molecule. The Man-PTS system is the receptor for leucocin A, as well as other Class IIa bacteriocins (Ramnath et al., 2000; Dalet et al., 2001; Diep et al., 2007) and is also used for uptake of glucose and fructose (Erni, 1989; Kotrba et al., 2001). Cells using the Man-PTS for metabolism and growth may not down regulate the Man-PTS even when class IIa bacteriocins are present (Dalet et al., 2001). In the current study, FSL R2-499, which is the most resistant to leucocin A, became sensitive to leucocin A when grown with mannose as the sole carbohydrate. However, this phenomenon was not observed for all strains. The variation in growth rates among strains can be attributed to strain individuality and carbohydrate preference. *L. monocytogenes* has different carbohydrate transport systems for uptake of sugars under normal conditions (Naghmouchi et al., 2006). Tessema et al. (2011) reported similar results for two spontaneously resistant strains of *L. monocytogenes* to sakacin P grown in mannose. The strains had difference growth rates in mannose despite both being resistant to sakacin P. Interestingly, the strain with resistance to a high bacteriocin concentration repressed *mptA* expression, while the strain with resistance to an intermediate level of bacteriocin overexpressed *mptA* despite have a reduced growth rate in mannose. Under situations where cells need to survive, for example in the presence of a bacteriocin, the preferred sugar transport system may change in response to carbohydrate availability. This may indicate that mechanisms other than carbohydrate transport systems are responsible for the development of resistance.

Glucose is the preferred carbohydrate of *L. monocytogenes* (Premaratne et al., 1991); however, strains used in this study had a faster growth rate in the presence of cellobiose and leucocin A. Cellobiose is transported across the cell membrane by the β-glucoside-PTS (Gravesen et al., 2000) and the lactose-PTS (Dalet et al., 2003). *L. monocytogenes* that have lost enzyme II subunit A/B or subunit C of the Man-PTS are resistant to class IIa bacteriocins (Dalet et al., 2001; Gravesen et al., 2002; Ramnath et al., 2004) and up-regulate the β-glucoside-PTS (Gravesen et al., 2000, 2002). This explains the shorter lag phase and faster growth rate when cultures were grown in cellobiose and treated with bacteriocins. The most resistant strain, FSL R2-499, grew fastest in cellobiose and had the shortest lag phase, indicating a preference for an alternative carbohydrate transport system.

Cultures treated with carnocyclin A had the slowest growth rates, indicating that carnocyclin A is more effective against *L. monocytogenes* than leucocin A. Carnocyclin A also increased the lag phase of *L. monocytogenes*, except in cultures grown in sucrose. In sucrose, carnocyclin A slowed the growth rate of strains and shortened the lag phase, compared to growth in other carbohydrates. There may be a fitness cost to *L. monocytogenes* associated with the use of metabolic pathways involved in the breakdown of disaccharides. The shorter lag phase indicates that although there may be an additional fitness cost to using sucrose as an energy source, the machinery to transport sucrose is probably not involved in bacteriocin docking.

The ability of *L. monocytogenes* to develop resistance to carnocyclin A was decreased when grown in the presence of glucose, fructose, or mannose as the lag phase was substantially extended; however, resistance developed and growth of cultures occurred within 72 h of incubation. The mode of action of carnocyclin A include formation of voltage-dependent anion selective channels; however, docking molecules that would support initial attachment of carnocyclin A to the cytoplasmic membrane have not been described (Gong et al., 2009). Gabrielsen et al. (2012) identified maltose ABC transporter as possible docking molecular for garvicin ML, a cyclic bacteriocin produced by *Lactococcus lactis*. Cells of *Lactococcus lactis* IL1403 that were grown in maltose containing media were 44 times more sensitive to garvicin ML than the same strain grown in glucose. The effect of carbohydrates on the resistance development of *Listeria* was similar in case of leucocin A and carnocyclin A, which may argue in favor of the existence of a carbohydrate-specific docking molecular for carnocyclin A. However, strains of nisin-resistant *L. monocytogenes*, which are also resistant to Class IIa bacteriocins (Crandall and Montville, 1998), have a less hydrophobic cell membrane compared to sensitive strains (Ming and Daeschel, 1993) and a more positive cell surface due to D-alanylation of teichoic acid and lysinilation of phospholipids (Vadyvaloo et al., 2004a; Jacquet et al., 2012). This change in cell surface properties could also explain the formation of resistance to carnocyclin A. It is possible that poor interactions between carnocyclin A, which is hydrophobic and cationic, and the cell membrane, decrease the formation of anion selective pores and increase resistance to the bacteriocin.

## Conclusion

Carbohydrate, bacteriocin type, and strain variation all impact the growth of *L. monocytogenes. L. monocytogenes* can develop resistance to leucocin A and carnocyclin A. Resistance of *L. monocytogenes* to carnocyclin A takes longer to form than resistance to leucocin A, but after extended lag phases all isolated subpopulations exhibit a stable-resistant phenotype to repeat exposure to the bacteriocins. Cellobiose and sucrose increased the ability of *L. monocytogenes* to form resistance compared to other carbohydrates, which could pose a food safety risk for industry, depending on the composition of the bacteriocin-containing product. It is important to note that not all strains behaved the same and therefore innate metabolic patterns and resistance may influence how *L. monocytogenes* reacts to the application of bacteriocins. Further research is needed to identify key mechanisms in *L. monocytogenes* that are involved in the development of resistance and if there is a general response to bacteriocins regardless of class or mode of action. The result of this study should cause concern to the food industry with regard to bacteriocin application regardless of bacteriocin class.

## Author Contributions

LM, DB, and MG designed the experiments that were conducted by DB. DB drafted and wrote the manuscript. All authors contributed to data analysis and interpretation and read and approved the final manuscript.

## Conflict of Interest Statement

The authors declare that the research was conducted in the absence of any commercial or financial relationships that could be construed as a potential conflict of interest.
