# Supplementary material for: The Effect of Carbohydrates and Bacteriocins on the Growth Kinetics and Resistance of Listeria monocytogenes
Source: Front Microbiol. 2018 Mar 1;9:347. doi: 10.3389/fmicb.2018.00347 (PMC5838005; doi:10.3389/fmicb.2018.00347)
Supplement: Supplementary file 1 [file Data_Sheet_1.PDF]

Supplementary Material

The Effect of Carbohydrates and Bacteriocins on the Growth Kinetics and Resistance of *Listeria monocytogenes*

Danielle R. Balay[1], Michael G. Gänzle[1], Lynn M. McMullen[1*]
*Corresponding author: lynn.mcmullen@ualberta.ca

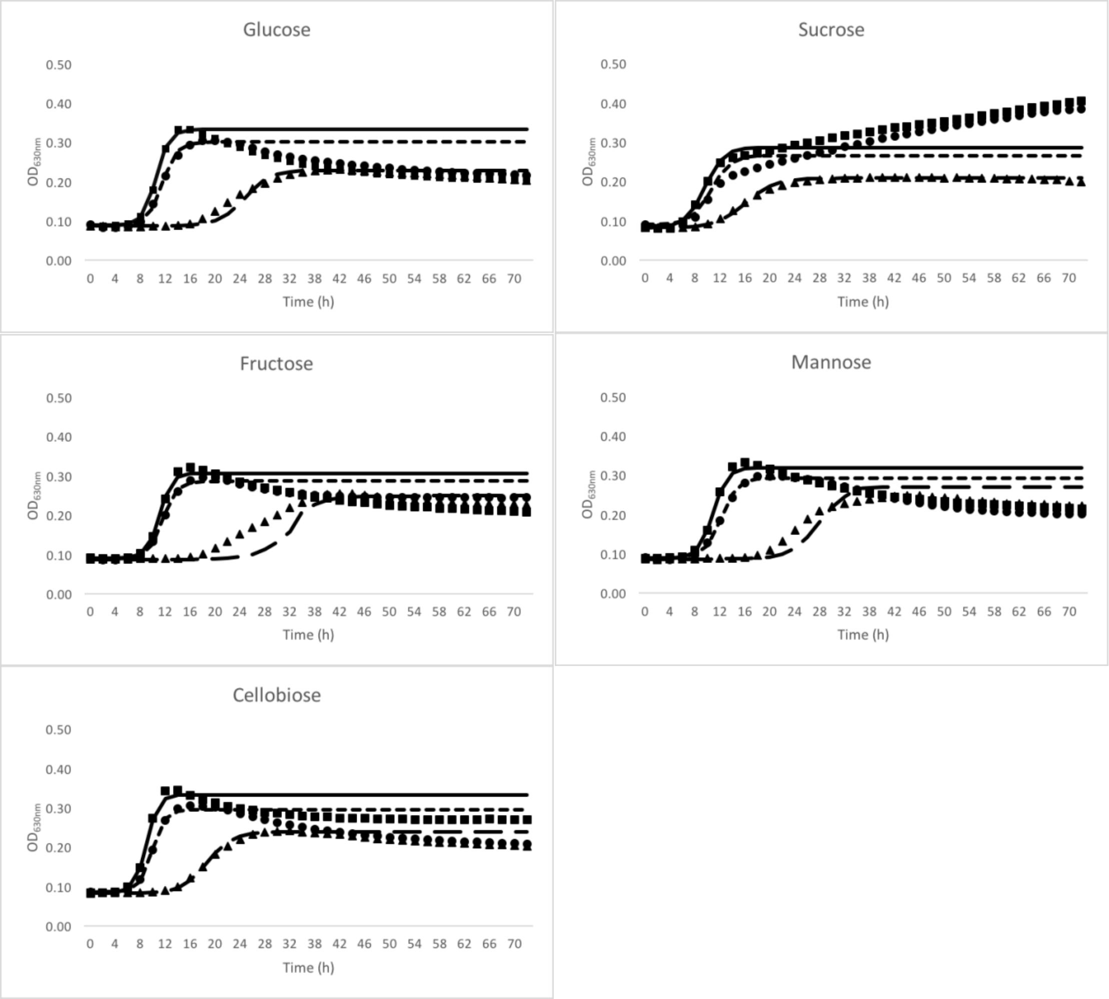

Figure S1 Experimental growth curve data (control, ■; treated with leucocin A, ●; treated with carnocyclin A, ▲) and modelled growth curve data (control, ——; treated with leucocin A, - - - ; treated with carnocyclin A, — -) for *L. monocytogenes* (FSL N1-227) grown in different carbohydrates at 25 °C. Optical density (630 nm) values displayed as the average of three independent replicates.

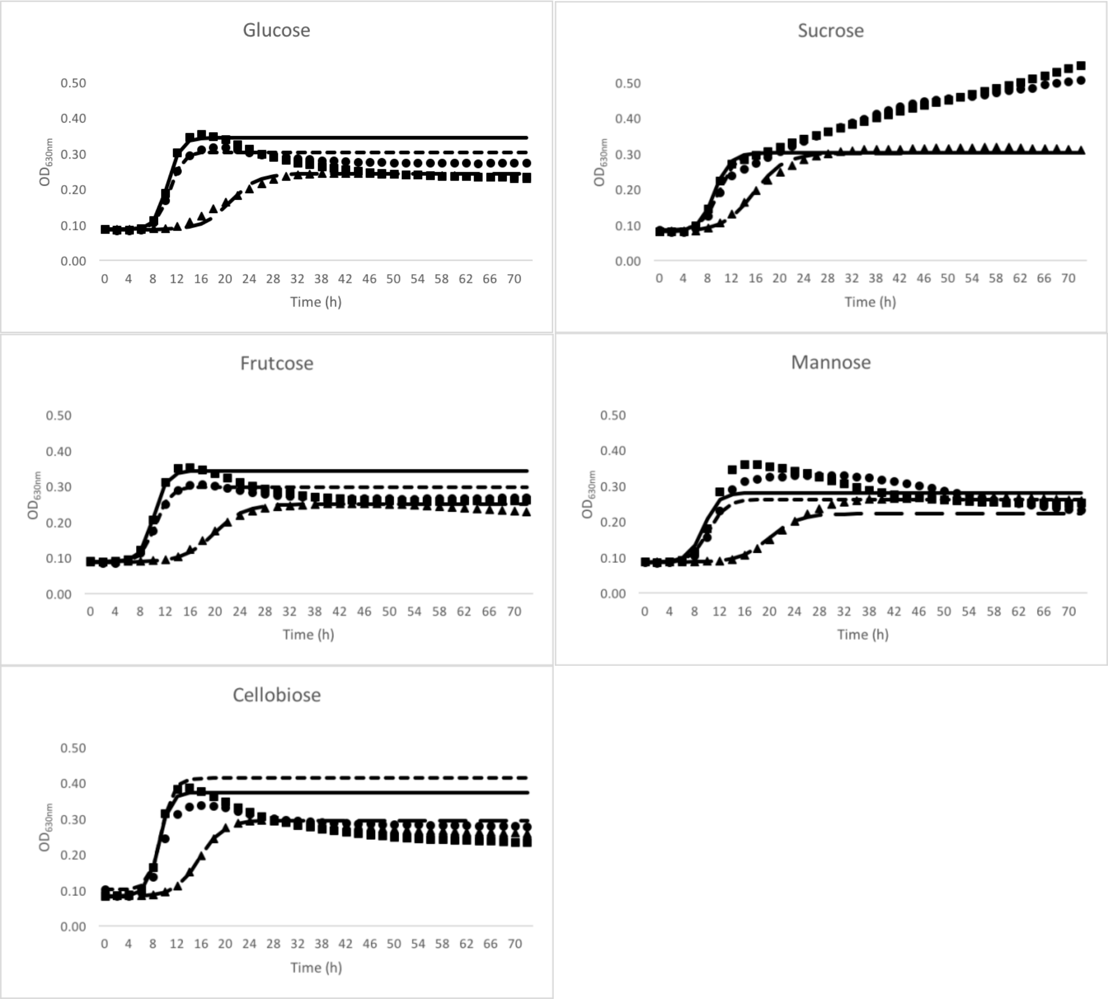

Figure S2 Experimental growth curve data (control, ■; treated with leucocin A, ●; treated with carnocyclin A, ▲) and modelled growth curve data (control, ——; treated with leucocin A, - - - ; treated with carnocyclin A, — -) for *L. monocytogenes* (FSL R2-499) grown in different carbohydrates at 25 °C. Optical density (630 nm) values displayed as the average of three independent replicates.

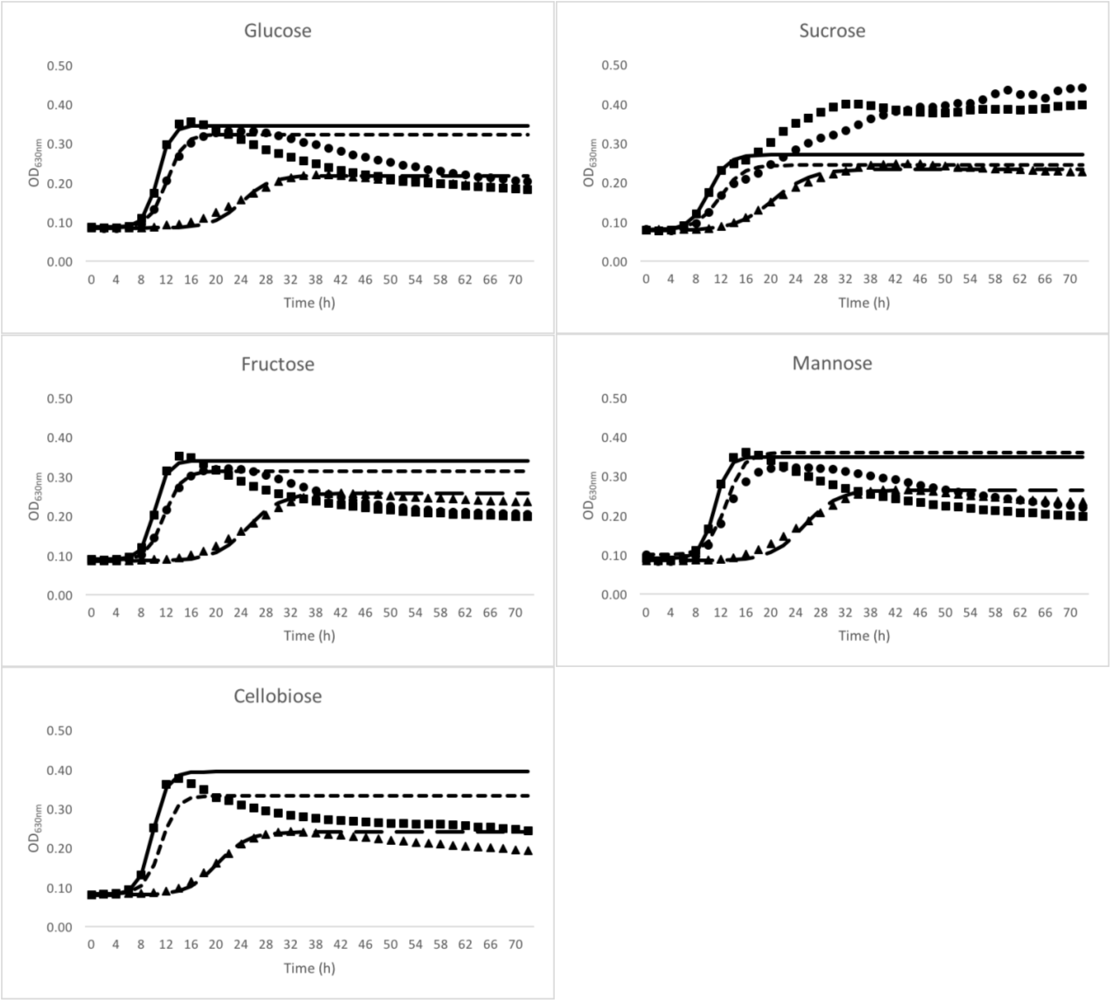

Figure S3 Experimental growth curve data (control, ■; treated with leucocin A, ●; treated with carnocyclin A, ▲) and modelled growth curve data (control, —— ; treated with leucocin A,--- ; treated with carnocyclin A,— -) for *L. monocytogenes* (FSL N3-013) grown in different carbohydrates at 25 °C. Optical density (630 nm) values displayed as the average of three independent replicates.

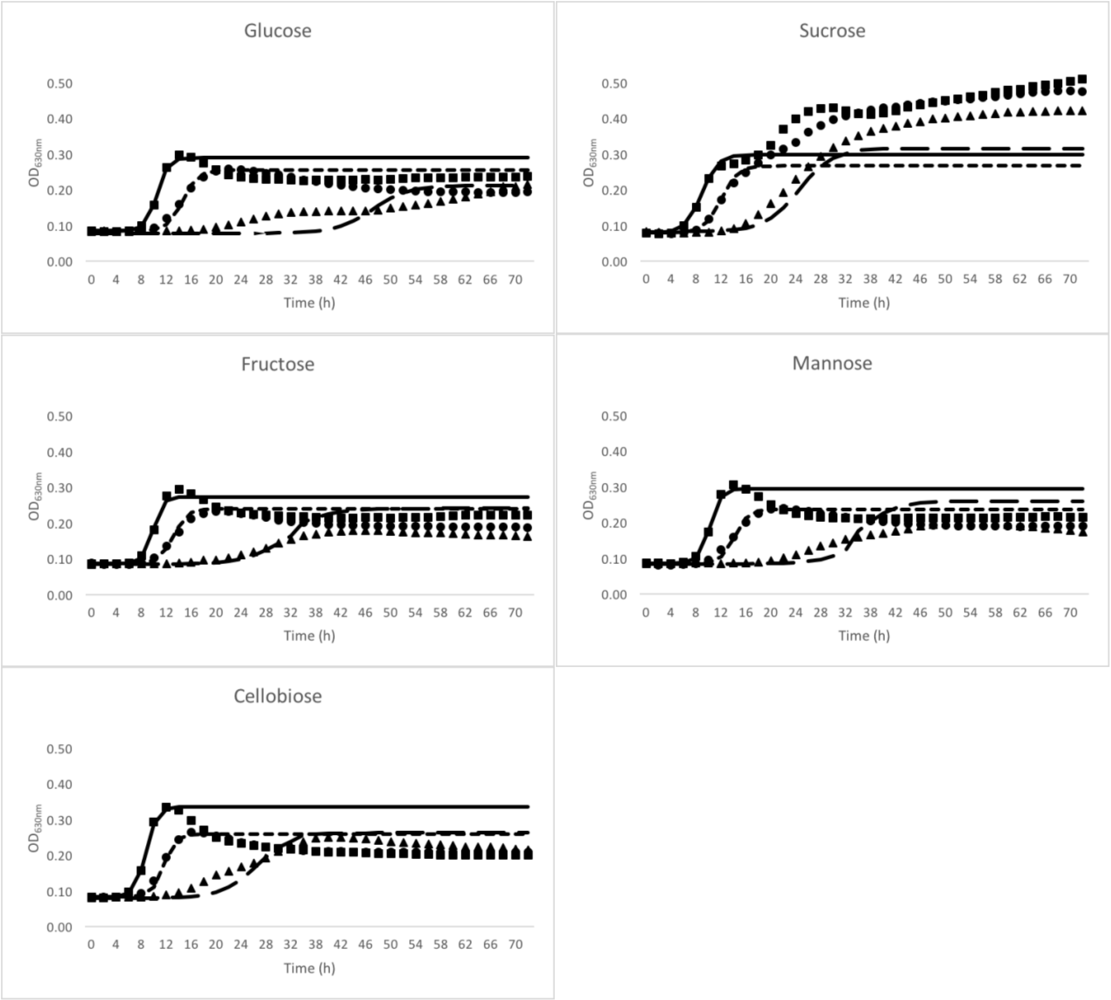

Figure S4 Experimental growth curve data (control, ■; treated with leucocin A, ●; treated with carnocyclin A, ▲) and modelled growth curve data (control, —— ; treated with leucocin A,--- ; treated with carnocyclin A,— -) for *L. monocytogenes* (FSL J1-177) grown in different carbohydrates at 25 °C. Optical density (630 nm) values displayed as the average of three independent replicates.

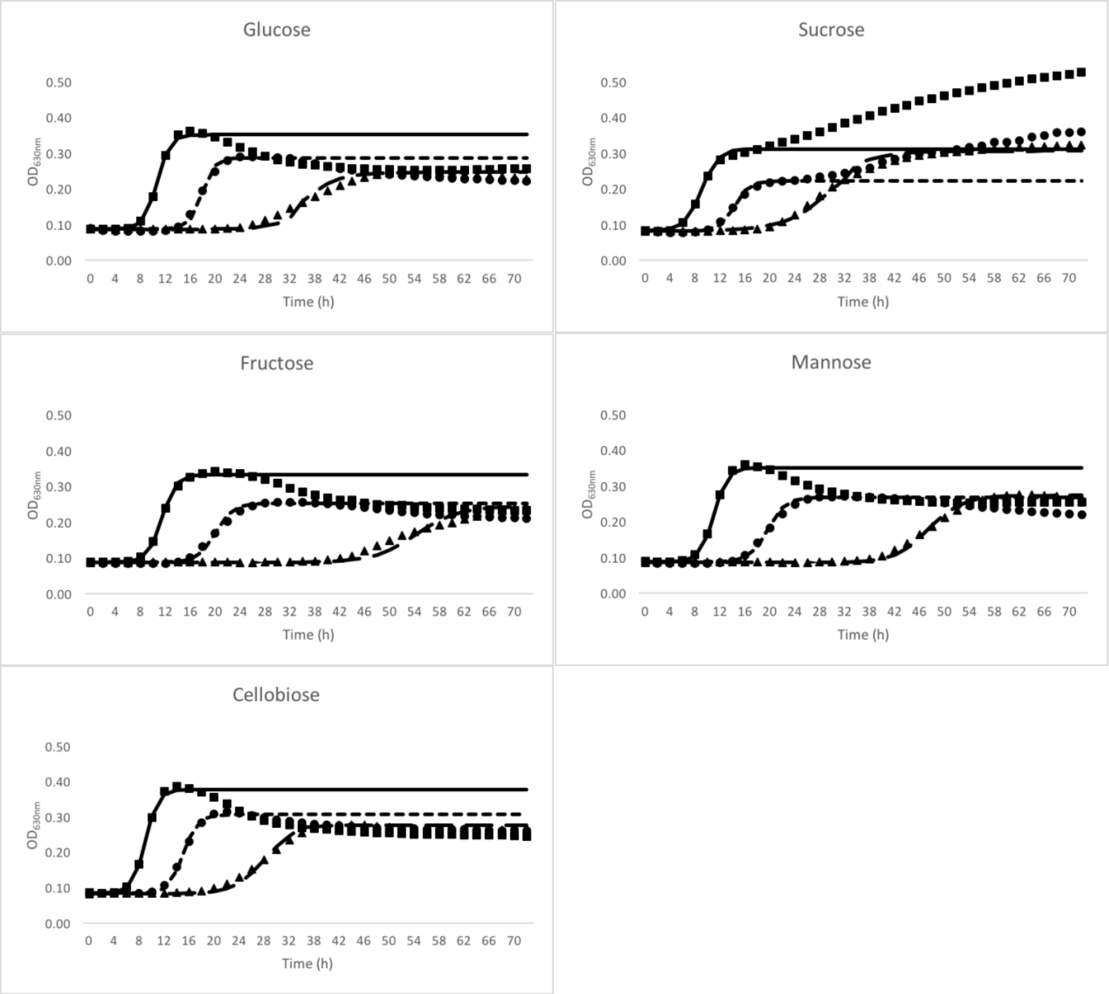

Figure S5 Experimental growth curve data (control, ■; treated with leucocin A, ●; treated with carnocyclin A, ▲) and modelled growth curve data (control, ——; treated with leucocin A,--- ; treated with carnocyclin A,— -) for *L. monocytogenes* (FSL C1-056) grown in different carbohydrates at 25 °C. Optical density (630 nm) values displayed as the average of three independent replicates.

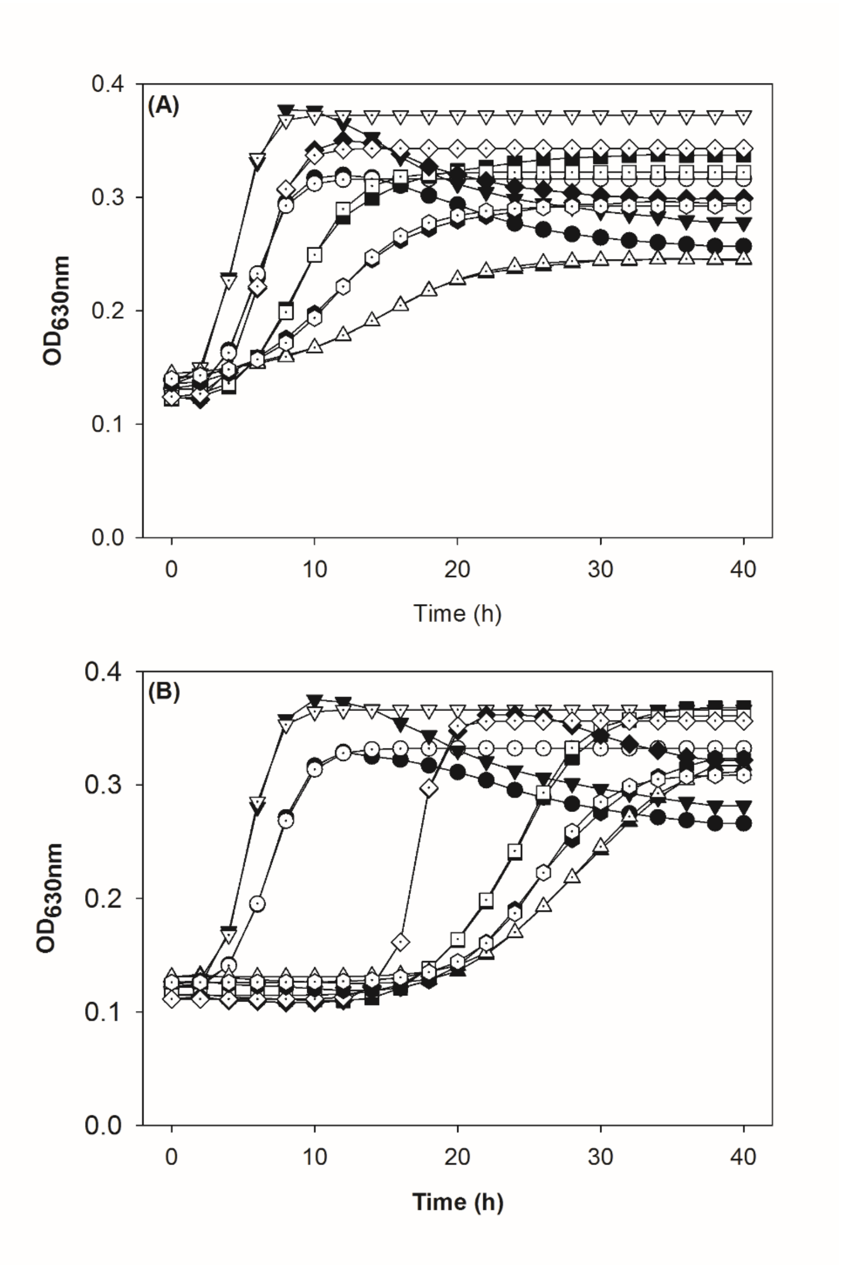

Figure S6 Experimental growth curve data (closed symbols) for original cultures of *L. monocytogenes* strains FSL R2-499 **(A)** and FSL C1-056 **(B)** compared to the modelled growth curve data (open symbols) grown in mannose (●,○); grown in mannose and treated with leucocin A (■,□); grown in mannose and treated with carnocyclin A (▲,△); grown in cellobiose (▼,▽); grown in cellobiose treated with leucocin A (◆,◇); grown in cellobiose treated with carnocyclin A (⬢,⬡) at 25 °C for 40 h. Optical density was measured at 630 nm and data is displayed as the average of four independent replicates.

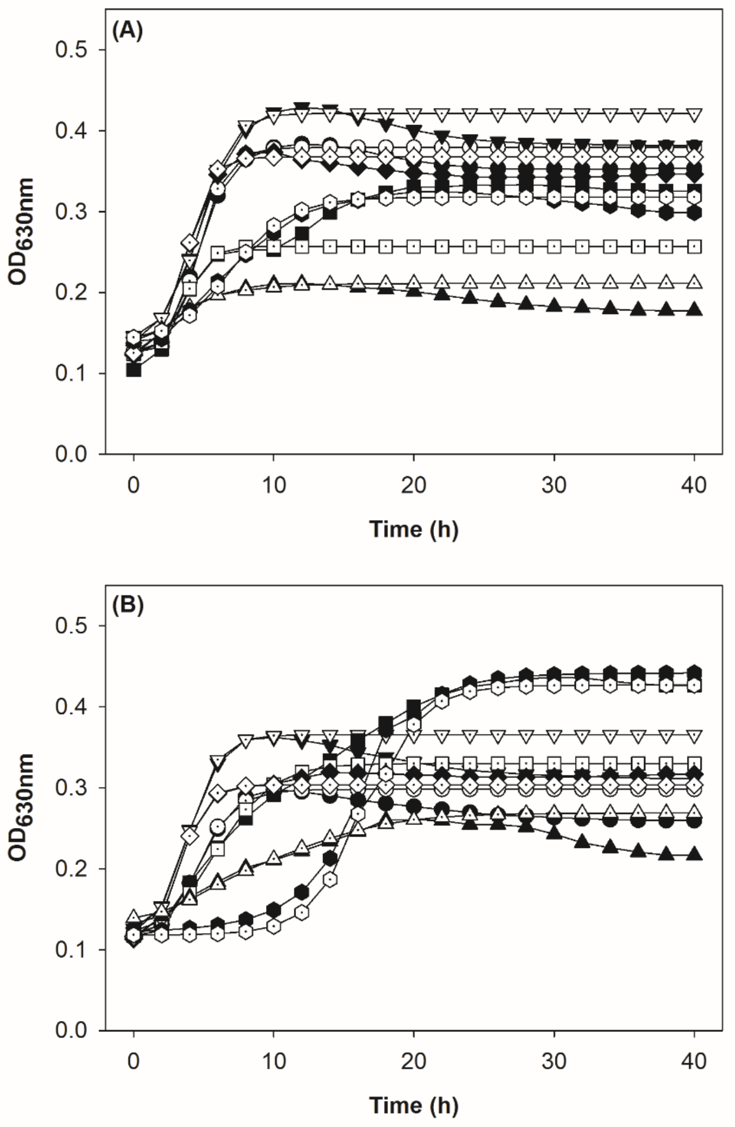

Figure S7 Experimental growth curve data (closed symbols) from isolated subpopulations from original cultures of *L. monocytogenes* strains FSL R2-499 **(A)** and FSL C1-056 **(B)** compared to the modelled growth curve data (open symbols) grown in mannose (●,○); grown in mannose and treated with leucocin A (■,□); grown in mannose and treated with carnocyclin A (▲,△); grown in cellobiose (▼,▽); grown in cellobiose treated with leucocin A (◆,◇); grown in cellobiose treated with carnocyclin A (⬢,⬡) at 25 °C for 40 h. Optical density was measured at 630 nm and data is displayed as the average of four independent replicates.

Table S1 Comparisons of maximal growth rate and lag phase of the original cultures and isolated subpopulations of *L. monocytogenes* determined at 25 °C in basal medium supplemented with mannose or cellobiose in the presence of 3.3 mM leucocin A or carnocyclin A (n=3).

			**original culture**		**subpopulation**	
			**μ_m***	**λ†**	**μ_m**	**λ**
**FSL C1-056**	**mannose**	**control**	0.22±0.02	3.85±0.55	0.23±0.07	2.04±0.35
		**leucocin A**	0.17±0.04	18.79±2.99	0.15±0.05	1.69±0.52
		**carnocyclin A**	0.10±0.02	23.43±4.20	0.07±0.01	1.76±1.62
	**cellobiose**	**control**	0.24±0.05	3.25±0.51	0.28±0.06	1.33±0.37
		**leucocin A**	0.45±0.21	15.00±1.36	0.30±0.11	1.38±0.28
		**carnocyclin A**	0.11±0.04	20.37±3.32	0.16±0.06	9.78±3.73
**FSL R2-499**	**mannose**	**control**	0.30±0.07	2.96±0.37	0.31±0.04	2.28±0.28
		**leucocin A**	0.14±0.04	4.20±1.07	0.23±0.13	1.86±1.33
		**carnocyclin A**	0.04±0.03	4.83±3.38	0.09±0.06	1.41±0.86
	**cellobiose**	**control**	0.33±0.04	2.39±0.81	0.25±0.09	1.68±0.99
		**leucocin A**	0.27±0.04	3.78±0.48	0.44±0.26	1.68±0.97
		**carnocyclin A**	0.07±0.05	3.98±1.97	0.11±0.02	2.31±0.80

* maximal growth rate (h^{-1}), † lag phase (h)
